# The Impact of a Crisis Resolution Home Treatment Team on Hospital Admission, Symptom severity and Service User Functioning over Five years

**DOI:** 10.1192/j.eurpsy.2023.1002

**Published:** 2023-07-19

**Authors:** S. Crowley, S. McDonagh, D. Carolan, K. O’Connor

**Affiliations:** 1 Psychiatry, North Lee Mental Health Services, Health Service Executive South, Ireland; 2 Psychiatry, South Lee Mental Health Services, Cork, Ireland

## Abstract

**Introduction:**

Crisis Resolution Home Treatment Teams (CRHTTs) offer short-term specialist psychiatric input to service users experiencing acute mental illness or crisis in the community. The South Lee CRHTT was setup in 2015.

**Objectives: Primary objectives:**

To evaluate the impact of treatment given by a CRHTT in terms of:

1. Preventing hospital admission,

2. Impact on service user’s symptoms and overall functioning

3. Service user’s satisfaction with the service

**Secondary Objectives:**

To evaluate patient characteristics of those attending the CRHTT, and to assess qualitative data provided by service users using thematic analysis.

**Methods:**

All the service users treated by South Lee CRHTT between 2016-2020 were included in this review. Standardized quantitative measures are routinely taken by the South Lee HBTT before and after treatment. The Brief Psychiatric Rating Scale (BPRS) was used to measure symptom reduction, and the Health of the Nation Outcome Scale (HONOS) was used to measure quality of life/health outcomes. The Client Satisfaction Questionnaire- version 8 (CSQ-8) was used to evaluate service user satisfaction quantitatively, and service users were also asked for qualitative data.

**Results:**

1041 service users were treated by the service, between 2016-2020. Treatment by the CRHTT was shown to be effective across all primary outcome measures. Inpatient admissions in the areas served by the CRHTT fell by 38.5% after its introduction. BPRS scores were reduced significantly (p<.001), from a mean score of 32.01 to 24.64 before and after treatment. Mean HoNOS scores were 13.6 before and 9.1 after treatment (p<.001). Of the 1041 service users receiving the CSQ-8, 180 returned it completed (17.3%). Service users’ median responses were “very positive” on a 4 point-Likert scale to all 8 items on the CSQ-8, and qualitative data were thematically analysed.

**Conclusions:**

CRHT was shown to be effective at preventing inpatient admission. CRHT was shown to be an effective option for the treatment of acute mental illness and crisis, using quantitative measures. Feedback gained from service users suggests that overall patient satisfaction with the CRHTT service was high.

**Disclosure of Interest:**

None Declared

